# Association Between Dysmenorrhea and Endometrial Cancer: A Mendelian Randomization Study

**DOI:** 10.1155/prm/4194108

**Published:** 2025-07-23

**Authors:** Qiuyuan Huang, Xizhen Huang, Liyuan Huang, Yanglin Wang, Suyu Li, Xiangqin Zheng

**Affiliations:** ^1^Department of Radiation Oncology, Fujian Maternity and Child Health Hospital, College of Clinical Medicine for Obstetrics & Gynecology and Pediatrics, Fujian Medical University, Fuzhou, Fujian, China; ^2^Department of Radiation Oncology, Fujian Children's Hospital (Fujian Branch of Shanghai Children's Medical Center), College of Clinical Medicine for Obstetrics & Gynecology and Pediatrics, Fujian Medical University, Fuzhou, Fujian, China; ^3^The Social Public Relations Section, Fujian Province Blood Center, Fuzhou, Fujian, China; ^4^Department of Gynecology, Fujian Maternity and Child Health Hospital, College of Clinical Medicine for Obstetrics & Gynecology and Pediatrics, Fujian Medical University, Fuzhou, Fujian, China

**Keywords:** causal inference, dysmenorrhea, endometrial cancer, Mendelian randomization, pain severity

## Abstract

**Background:** Dysmenorrhea is a common gynecological symptom among reproductive-aged women, associated with substantial pain and decreased quality of life. Previous studies have suggested that inflammatory and hormonal fluctuations linked to dysmenorrhea may influence endometrial cancer (EC) risk though causality remains uncertain. This study aimed to investigate potential causal relationships between dysmenorrhea (including pain severity, analgesic use, endometriosis, and related pelvic pain) and EC risk using a Mendelian randomization (MR) approach.

**Methods:** A two-sample MR analysis was conducted using genome-wide association study (GWAS) data, selecting single nucleotide polymorphisms (SNPs) significantly associated with dysmenorrhea to assess EC risk. Primary analysis was performed with the inverse-variance weighted (IVW) method, while weighted median and MR-Egger analyses were conducted to enhance robustness.

**Results:** The IVW analysis showed a significant inverse association between dysmenorrhea and EC risk (OR = 0.883; 95% CI: 0.794–0.983; and *p*=0.023), which remained significant after adjusting for confounders (OR = 0.868; 95% CI: 0.775–0.971; and *p*=0.0136). Sensitivity analyses supported this protective association. Other factors, including pain severity, analgesic use, endometriosis, and related pelvic pain, showed no significant association with EC.

**Conclusion:** This study indicates a potential inverse relationship between dysmenorrhea and EC risk. These findings provide novel causal evidence for understanding complex associations in female reproductive health, underscoring the need for further research on dysmenorrhea in EC prevention.

## 1. Introduction

Dysmenorrhea is one of the most prevalent gynecological symptoms among women of reproductive age, typically characterized by intense lower abdominal pain that significantly affects quality of life and work productivity [1, 2]. It is classified into primary and secondary types, with distinct etiologies and health implications. Globally, an estimated 50%–90% of women experience dysmenorrhea during their reproductive years, with notable variability in pain intensity and symptom presentation [3]. Primary dysmenorrhea is often associated with excessive secretion of inflammatory mediators such as prostaglandins [4], while secondary dysmenorrhea frequently co-occurs with underlying gynecological conditions, such as endometriosis, which is characterized by the ectopic growth of endometrial-like tissue that exacerbates menstrual pain [5].

Endometrial cancer (EC) is one of the most common malignancies of the female reproductive system, with established risk factors including obesity, diabetes, hypertension, nulliparity, early menarche, and late menopause [6]. While most observational studies have emphasized the potential adverse effects of chronic inflammation and hormonal imbalances associated with dysmenorrhea as contributors to EC risk, emerging evidence has also highlighted a possible protective role. The research suggests that frequent menstrual shedding and prostaglandin-induced endometrial turnover may help prevent the accumulation of abnormal endometrial cells, thereby potentially reducing the risk of EC [7, 8]. However, the association between dysmenorrhea and EC remains inconclusive. Some studies indicate that a robust menstrual response may promote endometrial health [9, 10] whereas others argue that the chronic inflammation and hormonal fluctuations inherent in dysmenorrhea could elevate EC risk [2, 11]. Therefore, investigating the potential causal relationship between dysmenorrhea and EC is of significant clinical importance.

Moreover, the severity of dysmenorrhea pain may reflect individual differences in endocrine and inflammatory states, and analgesic use is a common method for managing dysmenorrhea [12]. However, direct evidence linking pain severity and analgesic use with EC risk is currently insufficient and warrants further investigation [13]. In addition, endometriosis, a common cause of secondary dysmenorrhea, has been suggested as a potential risk factor for EC, though findings remain inconsistent, and a consensus has yet to be reached [14, 15]. Similarly, other pelvic pain symptoms related to the female reproductive organs may also contribute to the risk of EC, but existing research is limited regarding the specific impact of these factors [14].

Traditional observational studies face challenges in establishing causality due to confounding biases. To address these challenges, this study employed a Mendelian randomization (MR) approach using genome-wide association study (GWAS) data to investigate the causal relationship between dysmenorrhea (including menstruation quality of life impact, pain severity, analgesic use, endometriosis, and other related pain symptoms) and EC risk. MR leverages genetic variants as instrumental variables, effectively minimizing biases inherent in observational research. This study aims to provide novel causal evidence regarding the potential impact of dysmenorrhea and related factors on EC risk, thereby contributing to a deeper understanding of the complex associations within disorders of the female reproductive system [16, 17].

## 2. Methods

### 2.1. Study Design

**Figure 1:**
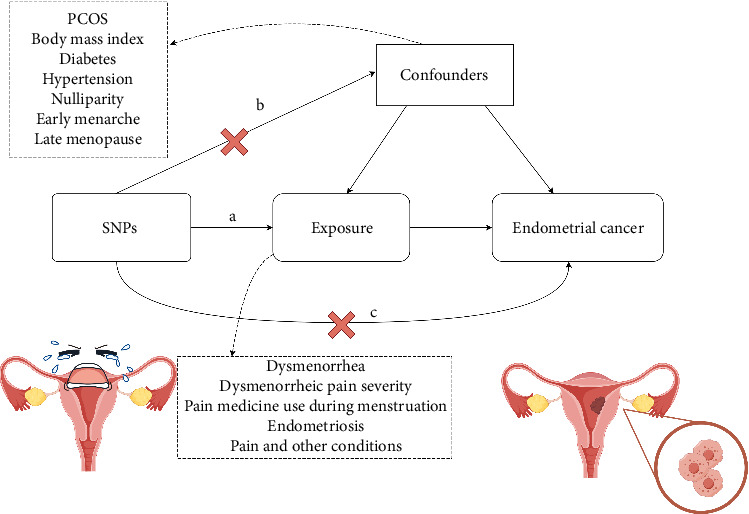
Three key assumptions of the Mendelian randomization study. (a) SNPs are strongly associated with exposure; (b) SNPs are independent of confounders; and (c) SNPs must only affect endometrial cancer and biomarkers via cheese intake. SNP: Single-nucleotide polymorphism.

This study was conducted in accordance with the principles outlined in the MR guideline [18]. This study utilized a two-sample MR design to investigate the causal relationship between genetic variations associated with dysmenorrhea and the risk of EC. MR analysis relied on three core assumptions: (a) selected single nucleotide polymorphisms (SNPs) are significantly associated with the exposure variables (dysmenorrhea and related traits); (b) SNPs are independent of known confounders; and (c) SNPs influence the outcome (EC) exclusively through the exposure variables, as illustrated in Figure 1.

### 2.2. Data Sources

Data for this study were sourced from publicly available GWAS databases. These datasets encompassed information on several aspects related to menstruation quality of life impact (dysmenorrhea) (*n* = 5734) [19], dysmenorrheic pain severity (*n* = 5734) [19], pain medicine use during menstruation (*n* = 5734) [19], endometriosis (*n* = 462,933), pain and other conditions associated with female genital organs and menstrual (*n* = 361,194), and EC (*n* = 240,027) [20]. This extensive data collection ensured a sufficient sample size and statistical power for drawing robust conclusions (Supporting Table 1). As the data were sourced from public databases with prior ethical approvals, no additional ethical review was required.

### 2.3. SNP Screening of Instrumental Variables

To comprehensively assess the effects of dysmenorrhea on EC risk, the study evaluated multiple dysmenorrhea-related traits. The primary outcome was the incidence of EC, derived from GWAS datasets related to EC. SNP selection and validation followed rigorous criteria to ensure the reliability of the MR analysis. Genome-wide significance (*p* < 5 × 10^−8^) was established to confirm strong associations between SNPs and dysmenorrhea-related factors. SNP independence was verified through pairwise linkage disequilibrium analysis, excluding SNPs exhibiting *R*^2^ > 0.001 (10,000 kb window) to minimize linkage disequilibrium bias. The strength of each SNP instrument was evaluated using the F-statistic, retaining only those with *F* > 10 to mitigate weak instrument bias. The F-statistic was calculated as *F* = (*R*^2^ × (K − *N* − 1))/((1 − *R*^2^) × K), where *R*^2^ is the proportion of variance explained by the SNPs, *N* is the sample size, and *K* is the number of SNPs.

### 2.4. Inverse-Variance Weighted (IVW) Method

To enhance analytical precision, adjustments were made for potential confounders, including body mass index (BMI), obesity, diabetes, hypertension, nulliparity, early menarche, and late menopause. To address potential confounders in causal inference, this study employed a two-stage SNP exclusion strategy, involving both separate exclusion and combined exclusion of SNPs associated with confounding factors. SNPs associated with confounding traits were excluded by querying the PhenoScanner V2 database. To ensure the robustness of our findings, we conducted both single and combined exclusions of these SNPs. These adjustments were crucial in reducing bias in estimating the causal relationship. In the main MR analysis, we employed an IVW meta-analysis using a random-effects model. In addition, sensitivity analyses utilizing weighted median and weighted mode methods were conducted to confirm the robustness of our findings.

### 2.5. Sensitivity Analyses

Several sensitivity analyses further ensured the reliability of our findings. The weighted median method provided robust estimates if over 50% of the information was derived from valid instrumental variables [21]. We employed MR-PRESSO to detect and remove horizontal pleiotropic outliers and assessed pleiotropy using MR-Egger regression intercepts. This step ensured that only valid instruments were used in the analysis. When significant outliers were identified, they were removed, and corrected estimates were obtained. These estimates were consistent with those from the IVW method, supporting the robustness of our findings. In addition, MR-Egger regression intercepts and heterogeneity tests (Cochran's Q) revealed no significant evidence of pleiotropy or heterogeneity, further validating our instrumental variables [22]. Finally, a leave-one-out analysis sequentially excluded individual SNPs to assess their influence on the results.

### 2.6. Statistical Analyses

All statistical analyses were conducted in R (Version 4.2.2) using the “TwoSampleMR” package, with a significance threshold set at *p* < 0.05. This rigorous methodological approach aimed to provide robust causal evidence on the relationship between dysmenorrhea-related factors and EC risk, addressing potential biases commonly encountered in observational studies.

## 3. Results

### 3.1. SNP Selection

The included studies, published between 2018 and 2021, met genome-wide significance criteria for the exposure factors, with all F-statistics exceeding 10 and *p* values below 5 × 10^−8^ (Supporting Tables 2–6). When the outcome was EC, SNPs associated with exposure variables and potential confounders were identified as follows: rs12543117, rs4687086, rs142412095, rs10808874, rs7775980, rs3094610, rs7766106, rs4876346, rs11031005, rs3174744, and rs61768001. No SNPs showed direct association with EC.

### 3.2. MR Results

**Figure 2:**
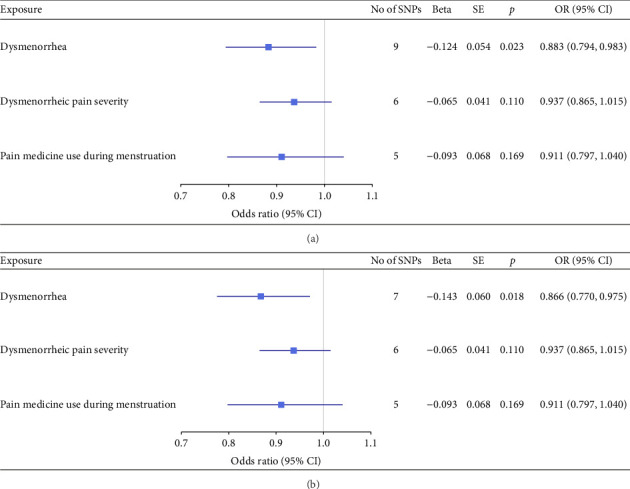
Associations of genetically predicted endometrial cancer with dysmenorrhea and related traits. (a) MR results before confounder adjustment. (b) MR results after confounder adjustment. CI: confidence interval; OR: odds ratio; SNP: single-nucleotide polymorphism fidence interval; OR: odds ratio; SNP: single-nucleotide polymorphism; SE: standard error.

Before adjusting for confounders, the IVW analysis revealed a significant inverse association between dysmenorrhea and EC risk (OR: 0.883; 95% CI: 0.794–0.983; and *p*=0.023; Figure 2(a) and Table 1). The exposure variables, including dysmenorrheic pain severity, pain medicine use during menstruation, endometriosis, pain and other conditions associated with female genital organs and menstrual, showed no significant association with EC risk (*p* > 0.05; Figure 2(a) and Supporting Table S7).

**Figure 3:**
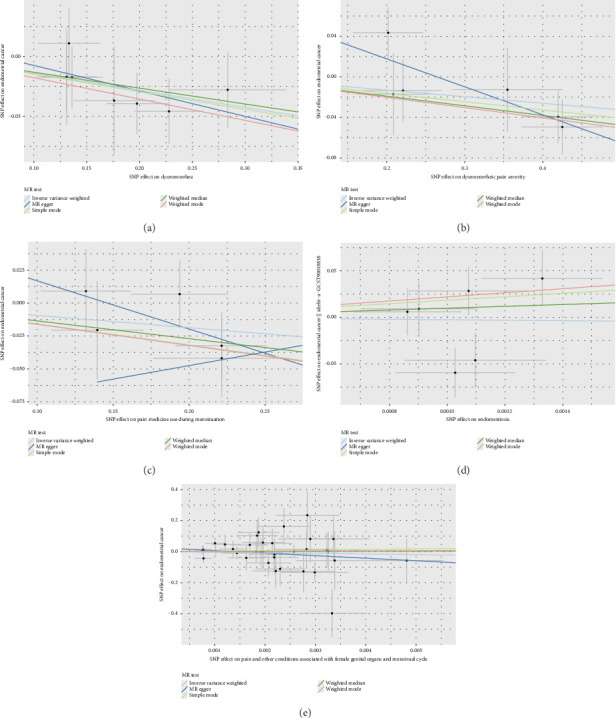
Scatter plot of the association of endometrial cancer with dysmenorrhea and related traits. (a) Dysmenorrhea; (b) dysmenorrheic pain severity; (c) pain medicine use during menstruation; (d) endometriosis; and (e) pain and other conditions associated with female genital organs and menstrual.Each black dot indicates a SNP, plotted by the estimate of SNP on individual endometrial cancer and the estimate of SNP on the risk of endometrial cancer with standard error bars. The slopes of the lines correspond to causal estimates using each of the different methods. SNP: single nucleotide polymorphism.

Multivariable two-sample MR analysis further evaluated the relationship between dysmenorrhea and EC risk. Regardless of the adjustment for confounders (including partial adjustments and those excluding SNPs associated with dysmenorrhea), IVW analysis consistently indicated a significant inverse association (OR: 0.866; 95% CI: 0.770–0.975; and *p*=0.018; Table 1 and Figures 2(b) and 3(a)). After adjusting for confounders, the IVW analysis indicated no significant associations between EC risk and exposure variables, including dysmenorrheic pain severity, pain medicine use during menstruation, endometriosis, and pain and other conditions associated with female genital organs and menstrual (*p* > 0.05; Figure 2(b), Supporting Table S7, and Figures 3(b), 3(c), 3(d), and 3(e)).

### 3.3. Sensitivity Analyses

To assess the robustness of the results, multiple sensitivity analyses were conducted both before and after adjusting for confounders. For dysmenorrhea and EC, the weighted median method produced *p* values ranging from 0.032 to 0.086, which aligned with the IVW results and indicated a suggestive trend (Table 1). MR-Egger regression yielded a *p* value of 0.363, indicating that horizontal pleiotropy did not significantly influence the results, further supporting the reliability of the IVW findings. In addition, the pleiotropy test (*p*=0.730) and heterogeneity test (*p*=0.910) showed no significant effects (Supporting Table 8 and Table 2).

For dysmenorrheic pain severity, IVW analysis showed no significant association with EC risk (*p* > 0.05). However, the weighted median method indicated a potential inverse trend (*p* < 0.05). MR-Egger analysis yielded *p* values between 0.05 and 0.1, suggesting a weak association that did not reach conventional significance. Both pleiotropy and heterogeneity tests had *p* values > 0.1, demonstrating no evidence of pleiotropy or heterogeneity, further validating the robustness of these findings (Supporting Table 8 and Table 2).

For pain medicine use during menstruation, endometriosis, pain, and other conditions associated with female genital organs and menstrual, neither the weighted median nor MR-Egger analyses demonstrated significant associations with EC risk (all *p* > 0.1). Pleiotropy and heterogeneity tests also showed no significant results (*p* > 0.1), confirming the absence of pleiotropy and heterogeneity issues in the instrumental variables (Supporting Tables 8 and Table 2). Additional sensitivity analyses, including leave-one-out analysis, forest plots, and funnel plots (Supporting Figures 1∼3), consistently supported these findings, demonstrating stability and robustness across the evaluated factors.

## 4. Discussion

This study presents the first systematic MR analysis to investigate the potential causal relationship between dysmenorrhea and EC. Our findings revealed a significant inverse association between dysmenorrhea and EC risk. However, factors related to dysmenorrhea, such as pain severity, analgesic use during menstruation, endometriosis, and other pain symptoms linked to female reproductive organs, showed no significant correlations with EC. These results provide novel insights into the complex interplay between menstrual health and gynecological cancer risk.

This study is the first to report an association between dysmenorrhea and EC risk, in contrast to previous research primarily focused on the relationship between secondary dysmenorrhea caused by conditions such as endometriosis and EC [5]. The inverse relationship between dysmenorrhea and EC risk persisted even after adjusting for potential confounders (OR = 0.868, 95% CI: 0.775–0.971, and *p*=0.0136). Dysmenorrhea is often characterized by painful uterine contractions accompanied by transient inflammatory responses during menstruation. It is hypothesized that this inflammatory environment, involving factors such as prostaglandins, tumor necrosis factor-alpha, and interleukin-6, may induce adaptive immune responses that enhance the endometrium's ability to resist malignant transformation [22]. Chronic, low-grade inflammation is known to play dual roles in cancer, either promoting or suppressing tumorigenesis depending on the context [23, 24]. Our findings suggest that dysmenorrhea may tip this balance toward enhanced immune surveillance, reducing the likelihood of abnormal cellular growth in the endometrium.

Hormonal regulation offers another plausible mechanism. Dysmenorrhea is associated with cyclical fluctuations in estrogen and progesterone levels, which are critical regulators of endometrial proliferation [25]. Prolonged exposure to unopposed estrogen is a well-established risk factor for EC, while the hormonal fluctuations seen in dysmenorrhea may contribute to a more balanced endocrine environment, potentially mitigating the carcinogenic effects of estrogen [26]. In addition, the frequent shedding and regeneration of the endometrial lining, typical in dysmenorrhea, may facilitate the removal of senescent or premalignant cells, reducing the risk of malignant transformation [26, 27]. Although these mechanisms are supported by existing theories, further biological and experimental studies are needed to validate them.

Interestingly, pain severity did not emerge as a significant factor in most analyses, indicating that the intensity of dysmenorrhea symptoms does not directly influence EC risk. While the weighted median method suggested a borderline significant trend (*p* < 0.05), MR-Egger analysis did not confirm this association. These findings imply that while dysmenorrhea itself may play a protective role, pain intensity is unlikely to act as an independent risk modifier. Future research could explore whether individual variations in hormonal or inflammatory responses mediate this subtle trend.

The absence of significant associations between EC and other dysmenorrhea-related factors, such as analgesic use or endometriosis, is also noteworthy. NSAIDs, commonly used to manage dysmenorrhea, possess anti-inflammatory properties [13], yet our results suggest no direct impact on EC risk. Similarly, while endometriosis has been implicated in increased risks for other gynecological cancers, such as ovarian cancer, its lack of association with EC in this study underscores the need for further exploration into its diverse pathophysiological effects [10, 15].

Sensitivity analyses reinforced the robustness of our findings. The inverse association between dysmenorrhea and EC was consistent across multiple MR methods, with no evidence of significant pleiotropy or heterogeneity. MR-Egger regression showed no horizontal pleiotropy (*p* > 0.1), and heterogeneity tests further supported the reliability of our results. These findings validate the strength of our instrumental variables and the causal inference drawn from this study.

Clinically, these results prompt a reevaluation of dysmenorrhea, often dismissed as a benign condition, in the context of long-term gynecological health. Although analgesics are effective for symptom relief, prolonged or frequent use may interfere with the protective inflammatory responses associated with dysmenorrhea. Management plans should aim to balance symptom control with the preservation of these natural protective mechanisms. Furthermore, integrating dysmenorrhea history into EC risk prediction models could enhance screening accuracy and open new avenues for early intervention.

We acknowledge that although we designed a bidirectional MR framework, we were unable to conduct a valid reverse MR analysis due to the limited number of genome-wide significant SNPs associated with EC, which restricted the construction of robust genetic instruments for EC as the exposure. In addition, primary dysmenorrhea commonly manifests during adolescence [28], while EC is predominantly diagnosed during the perimenopausal and postmenopausal periods [29]. Furthermore, EC is a multifactorial disease influenced by a wide range of genetic, hormonal, and environmental factors, complicating the interpretation of reverse causality.

While this study provides novel insights into the observed inverse association between genetically predicted dysmenorrhea and EC risk, several limitations should be acknowledged. First, the validity of MR relies on the strength and specificity of the instrumental variables. Although we applied stringent criteria to select genetic instruments and conducted multiple sensitivity analyses, the possibility of residual confounding and horizontal pleiotropy cannot be entirely excluded. Second, while we explicitly considered sample overlap between exposure and outcome GWAS datasets, the cross-ancestry design (European exposure data vs. Asian outcome data) introduces potential population stratification bias. Although consortia-level independence minimizes direct overlap risks, cryptic allelic heterogeneity due to ancestral differences may still affect instrument validity. Lastly, although our study suggests a potential inverse causal relationship, the underlying biological mechanisms linking dysmenorrhea to EC risk remain speculative. Future studies need to further validate the generalizability of the findings through the integration of multiple cohorts and functional experiments.

## 5. Conclusion

In conclusion, this study provides novel causal evidence for an inverse association between dysmenorrhea and EC, suggesting that dysmenorrhea may exert a protective effect against EC development. Although related factors such as pain severity, analgesic use, and endometriosis did not show significant associations, these findings offer a fresh perspective on the interplay between menstrual health and cancer risk. Future research should focus on elucidating the underlying biological mechanisms and integrating dysmenorrhea into comprehensive EC risk assessment and prevention strategies.

## Figures and Tables

**Table 1:** Associations between dysmenorrhea and endometrial cancer were evaluated using multivariable two-sample Mendelian randomization.

Method	Adjustment	*p*	OR (95% CI)
IVW	No	0.023	0.883 (0.794∼0.983)
MR-Egger		0.305	0.797 (0.534∼1.191)
Weighted median		0.071	0.878 (0.762∼1.011)

IVW	Obesity-related traits	0.014	0.868 (0.775∼0.971)
MR-Egger		0.340	0.809 (0.541∼1.209)
Weighted median		0.058	0.876 (0.764∼1.005)

IVW	BMI	0.030	0.884 (0.791∼0.988)
MR-Egger		0.230	0.780 (0.508∼1.198)
Weighted median		0.086	0.878 (0.756∼1.018)

IVW	Obesity-related traits, BMI	0.018	0.866 (0.770∼0.975)
MR-Egger		0.363	0.802 (0.520∼1.237)
Weighted median		0.032	0.878 (0.739∼0.986)

*Note:* rs10808874 was associated with obesity-related traits, and rs10989462 was correlated with BMI.

Abbreviations: BMI, body mass index; CI, confidence interval; IVW, inverse-variance weighted; MR, Mendelian randomization; OR, odds ratio.

**Table 2:** Associations between dysmenorrhea and endometrial cancer in sensitivity analyses using the weighted-median and MR-Egger methods after adjusting for confounding factors.

Exposure	Weighted median		MR-Egger		Pleiotropy		Heterogeneity	
	OR (95% CI)	*p*	OR (95% CI)	*p*	Intercept	*p*	Intercept	*p*
Dysmenorrhea	0.878 (0.739∼0.986)	0.032	0.802 (0.520∼1.237)	0.363	0.016	0.730	5	0.910
Dysmenorrheic pain severity	0.909 (0.831∼0.995)	0.038	0.759 (0.601∼0.958)	0.081	0.073	0.136	5	0.301
Pain medicine use during menstruation	0.874 (0.742∼1.028)	0.103	0.693 (0.343∼1.401)	0.382	0.054	0.495	4	0.711
Endometriosis	8.629e + 67 (0.033∼2.228E + 15)	0.106	2.410E + 25 (2.38E − 1∼12.43E + 61)	0.194	−0.054	0.294	12	0.046
Pain and other conditions	1.437 (4.20E − 9∼4.922E + 8)	0.971	2.310E − 07 (9.74E − 18∼5.476e + 3)	0.218	0.019	0.314	36	0.687

*Note:* Pain and others: pain and other conditions associated with female genital organs and menstrual cycle.

Abbreviations: CI, confidence interval; MR, Mendelian randomization; OR, odds ratio.

## Data Availability

The data that support the findings of this study are available in GWAS at https://gwas.mrcieu.ac.uk/, reference number ebi-a-GCST006636 and ebi-a-GCST90018838.

## Supporting Information

Supporting Table:

Supporting Table 1: The baseline characteristics of the exposure and outcome factors. SNPs: single-nucleotide polymorphisms.

Supporting Table 2: SNPs used as instrumental variables in the MR analyses of dysmenorrhea. Chr: chromosome; EA: effect allele; NEA: noneffect allele; SE: standard error; SNPs: single-nucleotide polymorphisms.

Supporting Table 3: SNPs used as instrumental variables in the MR analyses of dysmenorrheic pain severity. Chr: chromosome; EA: effect allele; NEA: noneffect allele; SE: standard error; SNP: single-nucleotide polymorphisms.

Supporting Table 4: SNPs used as instrumental variables in the MR analyses of pain medicine use during menstruation. Chr: chromosome; EA: effect allele; NEA: noneffect allele; SE: standard error; SNP: single-nucleotide polymorphisms.

Supporting Table 5: SNPs used as instrumental variables in the MR analyses of endometriosis. Chr: chromosome; EA: effect allele; NEA: noneffect allele; SE: standard error; SNP: single-nucleotide polymorphisms.

Supporting Table 6: SNPs used as instrumental variables in the MR analyses of pain and other conditions associated with female genital organs and menstrual cycle. Chr: chromosome; EA: effect allele; NEA: noneffect allele; SE: standard error; SNP: single-nucleotide polymorphisms.

Supporting Table 7: Associations of genetically predicted endometriosis/pain and other conditions with EC. SNP: single nucleotide polymorphism; Beta: begression coefficient; SE: standard error; OR: odds ratio; CI: confidence interval; pain and other conditions: pain and other conditions associated with female genital organs and menstrual cycle.

Supporting Table 8: Associations between dysmenorrhea and EC in sensitivity analyses using the weighted-median and MR-Egger methods before adjusting for confounding factors. CI: confidence interval; MR: Mendelian randomization; OR: odds ratio; pain and other conditions: pain and other conditions associated with female genital organs and menstrual cycle.

Supporting Figure:

Supporting Figure 1: Leave-one-out sensitivity analysis of the association of EC with dysmenorrhea and related traits. A: Dysmenorrhea; B: Dysmenorrheic pain severity; C: Pain medicine use during menstruation; D: Endometriosis; E: Pain and other conditions associated with female genital organs and menstrual. The dot and bar indicate the estimates and 95% confidence interval when the specific single nucleotide polymorphism is removed.

Supporting Figure 2: Forest plot of the association of EC with dysmenorrhea and related traits. A: Dysmenorrhea; B: Dysmenorrheic pain severity; C: Pain medicine use during menstruation; D: Endometriosis; E: Pain and other conditions associated with female genital organs and menstrual. The dot and bar indicate the causal estimate of individual diabetes on risks of EC.

Supporting Figure 3: Funnel plot of the association of EC with dysmenorrhea and related traits. A: Dysmenorrhea; B: Dysmenorrheic pain severity; C: Pain medicine use during menstruation; D: Endometriosis; E: Pain and other conditions associated with female genital organs and menstrual. Each black dot indicates a single nucleotide polymorphism.
