# Supplementary material for: Association Between Dysmenorrhea and Endometrial Cancer: A Mendelian Randomization Study
Source: Pain Res Manag. 2025 Jul 23;2025:4194108. doi: 10.1155/prm/4194108 (PMC12310317; doi:10.1155/prm/4194108)
Supplement: Supporting Information — Additional supporting information can be found online in the Supporting Information section. [file 4194108.f1.zip › Supplementary Figure.docx]

**Supplementary Figure :**


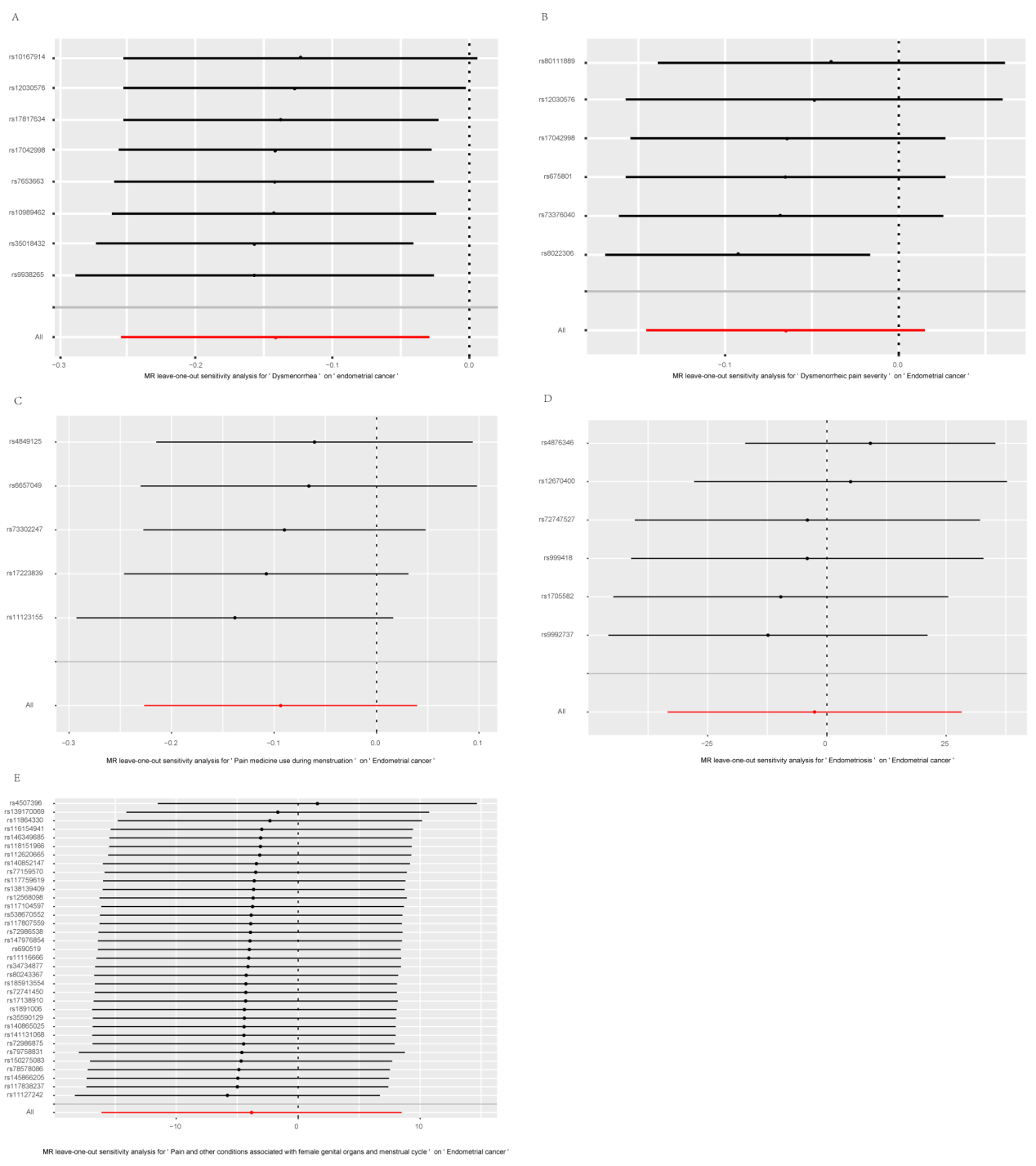


Supplementary Figure 1: Leave-one-out sensitivity analysis of the association of endometrial cancer with dysmenorrhea and related traits

A: Dysmenorrhea; B: Dysmenorrheic pain severity; C: Pain medicine use during menstruation; D: Endometriosis; E: Pain and other conditions associated with female genital organs and menstrual

The dot and bar indicate the estimates and 95% confidence interval when the specific single nucleotide polymorphism is removed.


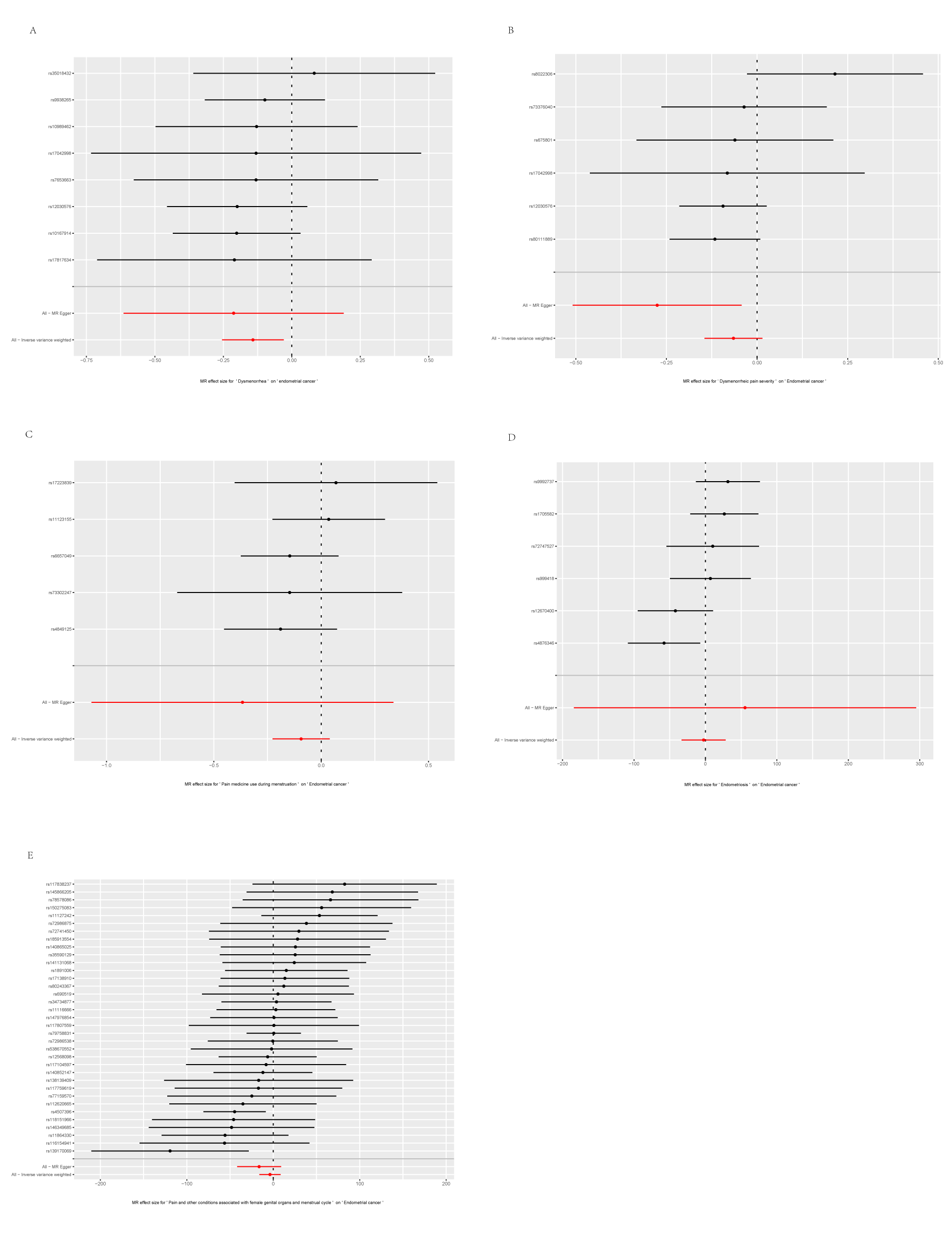


Supplementary Figure 2: Forest plot of the association of endometrial cancer with dysmenorrhea and related traits

A: Dysmenorrhea; B: Dysmenorrheic pain severity; C: Pain medicine use during menstruation; D: Endometriosis; E: Pain and other conditions associated with female genital organs and menstrual

The dot and bar indicate the causal estimate of individual diabetes on risks of endometrial cancer.


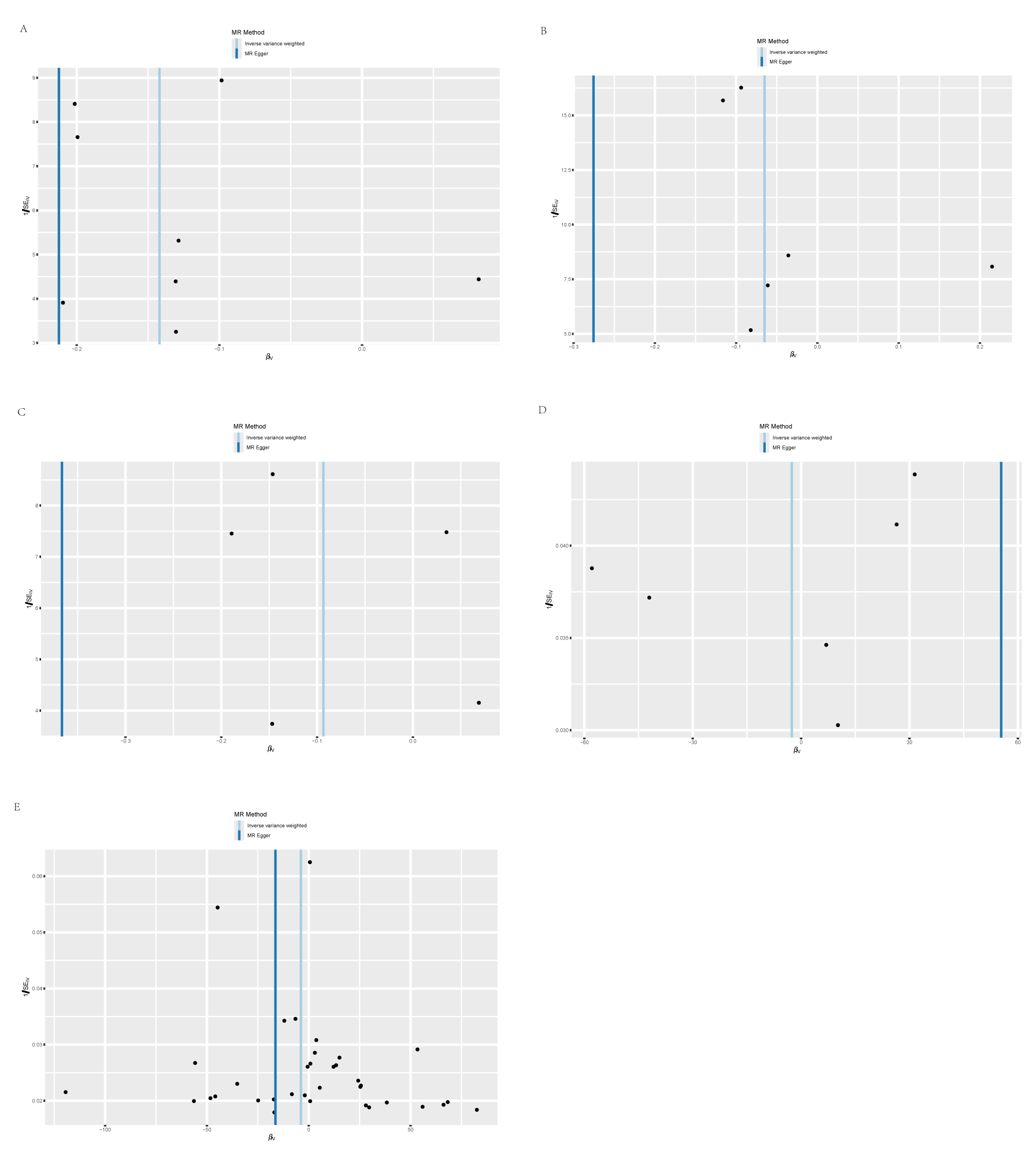


Supplementary Figure 3: Funnel plot of the association of endometrial cancer with dysmenorrhea and related traits.

A: Dysmenorrhea; B: Dysmenorrheic pain severity; C: Pain medicine use during menstruation; D: Endometriosis; E: Pain and other conditions associated with female genital organs and menstrual

Each black dot indicates a single nucleotide polymorphism.
