# Supplementary material for: Association Between Dysmenorrhea and Endometrial Cancer: A Mendelian Randomization Study
Source: Pain Res Manag. 2025 Jul 23;2025:4194108. doi: 10.1155/prm/4194108 (PMC12310317; doi:10.1155/prm/4194108)
Supplement: Supporting Information — Additional supporting information can be found online in the Supporting Information section. [file 4194108.f1.zip › Supplementary Table 1.docx]

Supplementary Table 1: The baseline characteristics of the exposure and outcome factors

| Trait | Year | Dataset | Population | Sample size | n case | n control | n SNP |
| --- | --- | --- | --- | --- | --- | --- | --- |
| Endometrial cancer | 2021 | ebi-a-GCST90018838 | European | 240,027 | 2,188 | 237,839 | 24,135,295 |
| Menstruation quality of life impact  (dysmenorrhea) | 2018 | ebi-a-GCST006636 | East Asian | 5,734 | 3,573 | 2,161 | 571,988 |
| Dysmenorrheic pain severity | 2018 | ebi-a-GCST006656 | East Asian | 5,734 | - | - | 571,989 |
| Pain medicine use during menstruation | 2018 | ebi-a-GCST006637 | East Asian | 5,734 | 1,813 | 3,921 | 571,987 |
| Endometriosis | 2018 | ukb-b-10903 | European | 462,933 | 3,809 | 459,124 | 9,851,867 |
| Pain and other conditions associated  with female genital organs and menstrual cycle | 2018 | ukb-d-N94 | European | 361,194 | 1,295 | 359,899 | 9,743,932 |

SNP: single-nucleotide polymorphisms
