# Supplementary material for: Association Between Dysmenorrhea and Endometrial Cancer: A Mendelian Randomization Study
Source: Pain Res Manag. 2025 Jul 23;2025:4194108. doi: 10.1155/prm/4194108 (PMC12310317; doi:10.1155/prm/4194108)
Supplement: Supporting Information — Additional supporting information can be found online in the Supporting Information section. [file 4194108.f1.zip › Supplementary Table 2.docx]

Supplementary Table 2: Single nucleotide polymorphisms used as instrumental variables in the mendelian randomization analyses of dysmenorrhea

| SNP | Chr | EA | NEA | Beta | SE | *p* | F |
| --- | --- | --- | --- | --- | --- | --- | --- |
| rs12030576 | 1 | T | G | -0.198 | 0.029 | 8.4879e-12 | 47 |
| rs17042998 | 2 | G | A | -0.131 | 0.029 | 4.60787e-06 | 21 |
| rs10167914 | 2 | G | A | -0.228 | 0.033 | 2.68658e-12 | 49 |
| rs7653663 | 3 | A | G | 0.131 | 0.028 | 3.6221e-06 | 21 |
| rs10808874 | 8 | C | T | -0.167 | 0.036 | 3.69267e-06 | 21 |
| rs35018432 | 8 | T | C | 0.133 | 0.029 | 5.88667e-06 | 21 |
| rs10989462 | 9 | G | A | 0.136 | 0.029 | 3.68477e-06 | 21 |
| rs17817634 | 12 | T | G | 0.176 | 0.038 | 4.83304e-06 | 21 |
| rs9938265 | 16 | G | A | -0.283 | 0.055 | 2.7077e-07 | 26 |

Chr: chromosome; EA: effect allele; NEA: non-effect allele; SE: standard error; SNP: single-nucleotide polymorphisms
