# Supplementary material for: Association Between Dysmenorrhea and Endometrial Cancer: A Mendelian Randomization Study
Source: Pain Res Manag. 2025 Jul 23;2025:4194108. doi: 10.1155/prm/4194108 (PMC12310317; doi:10.1155/prm/4194108)
Supplement: Supporting Information — Additional supporting information can be found online in the Supporting Information section. [file 4194108.f1.zip › Supplementary Table 3.docx]

Supplementary Table 3: Single nucleotide polymorphisms used as instrumental variables in the mendelian randomization analyses of dysmenorrheic pain severity

| SNP | Chr | EA | NEA | Beta | SE | *p* | F |
| --- | --- | --- | --- | --- | --- | --- | --- |
| rs12030576 | 1 | T | G | -0.420 | 0.046 | 1.13E-19 | 82 |
| rs80111889 | 2 | G | T | -0.425 | 0.052 | 1.90E-16 | 68 |
| rs17042998 | 2 | G | A | -0.208 | 0.046 | 6.09E-06 | 20 |
| rs675801 | 9 | A | G | 0.221 | 0.050 | 9.56E-06 | 20 |
| rs8022306 | 14 | A | G | 0.202 | 0.045 | 6.53E-06 | 20 |
| rs73376040 | 18 | T | G | -0.355 | 0.071 | 5.10E-07 | 25 |

Chr: chromosome; EA: effect allele; NEA: non-effect allele; SE: standard error; SNP: single-nucleotide polymorphisms
