# Supplementary material for: Association Between Dysmenorrhea and Endometrial Cancer: A Mendelian Randomization Study
Source: Pain Res Manag. 2025 Jul 23;2025:4194108. doi: 10.1155/prm/4194108 (PMC12310317; doi:10.1155/prm/4194108)
Supplement: Supporting Information — Additional supporting information can be found online in the Supporting Information section. [file 4194108.f1.zip › Supplementary Table 4.docx]

Supplementary Table 4: Single nucleotide polymorphisms used as instrumental variables in the mendelian randomization analyses of pain medicine use during menstruation

| SNP | Chr | EA | NEA | Beta | SE | *p* | F |
| --- | --- | --- | --- | --- | --- | --- | --- |
| rs6657049 | 1 | A | G | -0.221 | 0.030 | 2.231e-13 | 54 |
| rs11123155 | 2 | T | C | -0.194 | 0.032 | 1.53978e-09 | 36 |
| rs4849125 | 2 | A | G | -0.221 | 0.045 | 8.65765e-07 | 24 |
| rs73302247 | 7 | T | C | -0.140 | 0.030 | 3.77398e-06 | 21 |
| rs17223839 | 14 | G | A | -0.132 | 0.030 | 9.18058e-06 | 20 |

Chr: chromosome; EA: effect allele; NEA: non-effect allele; SE: standard error; SNP: single-nucleotide polymorphisms
