# Supplementary material for: Association Between Dysmenorrhea and Endometrial Cancer: A Mendelian Randomization Study
Source: Pain Res Manag. 2025 Jul 23;2025:4194108. doi: 10.1155/prm/4194108 (PMC12310317; doi:10.1155/prm/4194108)
Supplement: Supporting Information — Additional supporting information can be found online in the Supporting Information section. [file 4194108.f1.zip › Supplementary Table 5.docx]

Supplementary Table 5: Single nucleotide polymorphisms used as instrumental variables in the mendelian randomization analyses of endometriosis

| SNP | Chr | EA | NEA | Beta | SE | *p* | F |
| --- | --- | --- | --- | --- | --- | --- | --- |
| rs999418 | 1 | C | T | 0.0009 | 0.0002 | 4.20001e-06 | 21 |
| rs61768001 | 1 | C | T | 0.0018 | 0.0003 | 1e-11 | 46 |
| rs1705582 | 3 | A | G | -0.0011 | 0.0002 | 2.39999e-07 | 27 |
| rs9992737 | 4 | T | C | -0.0013 | 0.0002 | 2e-10 | 40 |
| rs72747527 | 5 | T | C | 0.0009 | 0.0002 | 7.59994e-06 | 20 |
| rs7775980 | 6 | G | A | 0.0013 | 0.0002 | 1.40001e-07 | 28 |
| rs3094610 | 6 | C | G | -0.0012 | 0.0003 | 4.39997e-06 | 21 |
| rs7766106 | 6 | T | C | 0.0009 | 0.0002 | 5.99998e-07 | 25 |
| rs12670400 | 7 | T | C | -0.0011 | 0.0002 | 7.19996e-06 | 20 |
| rs4876346 | 8 | T | C | -0.0010 | 0.0002 | 6.80002e-07 | 25 |
| rs11031005 | 11 | C | T | -0.0016 | 0.0003 | 1.5e-09 | 37 |
| rs3174744 | 17 | G | A | -0.0010 | 0.0002 | 2.19999e-06 | 22 |
| rs2918299 | 19 | T | C | 0.0013 | 0.0003 | 4e-07 | 26 |

Chr: chromosome; EA: effect allele; NEA: non-effect allele; SE: standard error; SNP: single-nucleotide polymorphisms
