# Supplementary material for: Association Between Dysmenorrhea and Endometrial Cancer: A Mendelian Randomization Study
Source: Pain Res Manag. 2025 Jul 23;2025:4194108. doi: 10.1155/prm/4194108 (PMC12310317; doi:10.1155/prm/4194108)
Supplement: Supporting Information — Additional supporting information can be found online in the Supporting Information section. [file 4194108.f1.zip › Supplementary Table 6.docx]

Supplementary Table 6: Single nucleotide polymorphisms used as instrumental variables in the mendelian randomization analyses of pain and other conditions associated with female genital organs and menstrual cycle

| SNP | Chr | EA | NEA | Beta | SE | *p* | F |
| --- | --- | --- | --- | --- | --- | --- | --- |
| rs72741450 | 1 | G | A | 0.0020 | 0.0004 | 2.45172e-06 | 22 |
| rs145866205 | 1 | A | G | 0.0024 | 0.0005 | 7.4535e-06 | 20 |
| rs12568098 | 1 | T | C | 0.0014 | 0.0003 | 6.69823e-06 | 20 |
| rs147976854 | 1 | T | C | 0.0033 | 0.0007 | 2.79692e-06 | 22 |
| rs140852147 | 2 | C | T | 0.0048 | 0.0007 | 3.71878e-11 | 44 |
| rs139170069 | 2 | A | C | 0.0033 | 0.0006 | 2.1731e-07 | 27 |
| rs11127242 | 2 | T | G | -0.0010 | 0.0002 | 1.40217e-07 | 28 |
| rs112620665 | 2 | G | A | 0.0021 | 0.0004 | 2.68065e-06 | 22 |
| rs72986538 | 2 | T | C | 0.0018 | 0.0004 | 9.6634e-06 | 20 |
| rs146349685 | 3 | A | G | 0.0023 | 0.0005 | 3.13422e-06 | 22 |
| rs4687086 | 3 | G | A | -0.0007 | 0.0001 | 4.29685e-06 | 21 |
| rs116154941 | 4 | C | T | 0.0022 | 0.0005 | 3.03326e-06 | 22 |
| rs140865025 | 4 | C | A | 0.0017 | 0.0004 | 3.29807e-06 | 22 |
| rs150275083 | 4 | C | A | 0.0018 | 0.0004 | 6.21112e-06 | 20 |
| rs4507396 | 4 | G | T | 0.0030 | 0.0007 | 8.2448e-06 | 20 |
| rs141131068 | 5 | C | T | 0.0034 | 0.0007 | 3.46298e-06 | 22 |
| rs538670552 | 6 | A | G | 0.0019 | 0.0004 | 9.58164e-06 | 20 |
| rs80243367 | 6 | C | G | 0.0014 | 0.0003 | 1.19168e-06 | 24 |
| rs117104597 | 7 | A | G | 0.0022 | 0.0005 | 8.03526e-06 | 20 |
| rs531512581 | 7 | C | A | 0.0030 | 0.0006 | 2.34628e-06 | 22 |
| rs78578086 | 7 | T | C | 0.0019 | 0.0004 | 3.94984e-06 | 21 |
| rs117759619 | 8 | T | C | 0.0022 | 0.0005 | 6.70301e-06 | 20 |
| rs118151966 | 8 | T | C | 0.0028 | 0.0006 | 6.22802e-06 | 20 |
| rs12543117 | 8 | C | G | -0.0006 | 0.0001 | 5.5538e-06 | 21 |
| rs185913554 | 9 | A | T | 0.0029 | 0.0006 | 5.83136e-06 | 21 |
| rs35590129 | 9 | G | A | 0.0021 | 0.0004 | 6.50894e-08 | 29 |
| rs17138910 | 10 | C | T | -0.0008 | 0.0002 | 8.86258e-06 | 20 |
| rs142412095 | 11 | C | T | 0.0025 | 0.0006 | 6.6819e-06 | 20 |
| rs690519 | 11 | C | G | -0.0028 | 0.0006 | 7.1791e-06 | 20 |
| rs11116666 | 12 | C | T | 0.0032 | 0.0007 | 1.49924e-06 | 23 |
| rs79758831 | 12 | T | C | 0.0026 | 0.0006 | 3.48097e-06 | 22 |
| rs138139409 | 12 | T | C | 0.0034 | 0.0008 | 8.98669e-06 | 20 |
| rs1891006 | 13 | T | G | -0.0008 | 0.0002 | 4.03562e-06 | 21 |
| rs117838237 | 15 | T | C | 0.0028 | 0.0006 | 7.84061e-06 | 20 |
| rs179771 | 16 | C | G | -0.0006 | 0.0001 | 7.50966e-06 | 20 |
| rs11864330 | 16 | A | G | -0.0008 | 0.0002 | 6.80989e-06 | 20 |
| rs77159570 | 17 | G | T | 0.0016 | 0.0004 | 8.20333e-06 | 20 |
| rs117807559 | 19 | A | G | 0.0020 | 0.0004 | 2.67979e-06 | 22 |
| rs72986875 | 19 | C | T | 0.0012 | 0.0003 | 5.15929e-06 | 21 |
| rs34734877 | 20 | G | T | 0.0010 | 0.0002 | 5.34404e-07 | 25 |

Chr: chromosome; EA: effect allele; NEA: non-effect allele; SE: standard error; SNP: single-nucleotide polymorphisms
