# Supplementary material for: Association Between Dysmenorrhea and Endometrial Cancer: A Mendelian Randomization Study
Source: Pain Res Manag. 2025 Jul 23;2025:4194108. doi: 10.1155/prm/4194108 (PMC12310317; doi:10.1155/prm/4194108)
Supplement: Supporting Information — Additional supporting information can be found online in the Supporting Information section. [file 4194108.f1.zip › Supplementary Table 7.docx]

Supplementary Table 7: Associations of genetically predicted endometriosis / Pain and other conditions with endometrial cancer

|  | Exposure | SNP | Beta | SE | *P* | OR (95% CI) |
| --- | --- | --- | --- | --- | --- | --- |
| Before adjusting for  confounding factors | Endometriosis | 13 | 12.925 | 9.240 | 0.162 | 4.106E+5 (5.596E-3~3.013E+13) |
|  | Pain and other conditions | 37 | -4.521 | 6.141 | 0.462 | 0.011(6.45E-08~1.835E+3) |
| After adjusting for  confounding factors | Endometriosis | 6 | -2.575 | 15.733 | 0.870 | 0.076(3.090E-15~1.878E+12) |
|  | Pain and other conditions | 35 | -3.823 | 6.274 | 0.542 | 0.022(9.97E-08~4.792E+3) |

SNP: single nucleotide polymorphism; Beta: begression coefficient; SE: standard error; OR: odds ratio; CI: confidence interval;Pain and other conditions: pain and other conditions associated with female genital organs and menstrual cycle
