# Supplementary material for: Association Between Dysmenorrhea and Endometrial Cancer: A Mendelian Randomization Study
Source: Pain Res Manag. 2025 Jul 23;2025:4194108. doi: 10.1155/prm/4194108 (PMC12310317; doi:10.1155/prm/4194108)
Supplement: Supporting Information — Additional supporting information can be found online in the Supporting Information section. [file 4194108.f1.zip › Supplementary Table 8.docx]

Supplementary Table 8: Associations between dysmenorrhea and endometrial cancer in sensitivity analyses using the weighted-median and MR-Egger methods before adjusting for confounding factors

| Exposure | Weighted Median |  | MR-Egger |  | Pleiotropy |  | Heterogeneity |  |
| --- | --- | --- | --- | --- | --- | --- | --- | --- |
|  | OR (95%CI) | *p* | OR (95%CI) | *p* | Intercept | *p* | Intercept | *p* |
| Dysmenorrhea | 0.878(0.762~1.011) | 0.071 | 0.797(0.534~1.191) | 0.305 | 0.02 | 0.619 | 8 | 0.956 |
| Dysmenorrheic pain severity | 0.909(0.831~0.995) | 0.038 | 0.759(0.601~0.958) | 0.081 | 0.073 | 0.136 | 5 | 0.301 |
| Pain medicine use during menstruation | 0.874(0.742~1.028) | 0.103 | 0.693 (0.343~1.401) | 0.382 | 0.054 | 0.495 | 4 | 0.711 |
| Endometriosis | 8.629e+67(0.033~2.228E+15) | 0.106 | 2.41E+25(2.38E~112.43E+61) | 0.194 | -0.054 | 0.294 | 12 | 0.046 |
| Pain and other conditions | 1.437(4.20E-9~4.922E+8) | 0.971 | 2.31E-07(9.74E-18~5.476e+3) | 0.218 | 0.019 | 0.314 | 36 | 0.687 |

CI: confidence interval; MR: Mendelian randomization; OR: odds ratio; Pain and other conditions: pain and other conditions associated with female genital organs and menstrual cycle
